# Recruiting and Retaining Youth and Young Adults in the Policy and Communication Evaluation (PACE) Vermont Study: Randomized Controlled Trial of Participant Compensation

**DOI:** 10.2196/18446

**Published:** 2020-07-20

**Authors:** Andrea C Villanti, Christie P Vallencourt, Julia C West, Catherine Peasley-Miklus, S Elisha LePine, Caitlin McCluskey, Elias Klemperer, Jeffrey S Priest, Alison Logan, Bill Patton, Nancy Erickson, Jennifer Hicks, Kathleen Horton, Shayla Livingston, Maria Roemhildt, Erin Singer, Megan Trutor, Rhonda Williams

**Affiliations:** 1 Department of Psychiatry Vermont Center on Behavior and Health University of Vermont Larner College of Medicine Burlington, VT United States; 2 Health Promotion & Disease Prevention Vermont Department of Health Burlington, VT United States; 3 Biomedical Statistics Research Core University of Vermont Burlington, VT United States; 4 Hark Inc Burlington, VT United States; 5 Communication Vermont Department of Health Burlington, VT United States; 6 Health Surveillance Vermont Department of Health Burlington, VT United States; 7 Public Health Policy Vermont Department of Health Burlington, VT United States; 8 Alcohol & Drug Abuse Programs Vermont Department of Health Burlington, VT United States

**Keywords:** recruitment, retention, adolescents, young adults, prevention

## Abstract

**Background:**

The standard approach for evaluating the effects of population-level substance use prevention efforts on youth and young adult perceptions and behaviors has been to compare outcomes across states using national surveillance data. Novel surveillance methods that follow individuals over shorter time intervals and capture awareness of substance use prevention policy and communication efforts may provide a stronger basis for their evaluation than annual cross-sectional studies.

**Objective:**

This study aimed to identify a combination of strategies to recruit a sample of youth and young adults sufficiently representative of the Vermont population and determine how best to retain a web-based panel of youth and young adults over a 6-month period.

**Methods:**

Eligible participants were Vermont residents aged 12 to 25 years who were willing to complete three 10 to 15-minute web-based surveys over a 6-month period. Recruitment was conducted via the following three main mechanisms: (1) web-based recruitment (paid and unpaid), (2) community-based recruitment through partners, and (3) participant referrals via a personalized link. Upon completion of the baseline survey, participants were randomly assigned to one of the following three retention incentive conditions: (1) guaranteed incentive (US $10), (2) lottery incentive (US $50 weekly lottery drawing), and (3) preferred method (guaranteed or lottery). Analyses examined cost per survey start by recruitment source, distribution of demographic characteristics across incentive conditions, and retention by study condition at 3-month and 6-month follow-ups.

**Results:**

Over a 10-week period in 2019, we recruited 480 eligible youth (aged 12-17 years) and 1037 eligible young adults (aged 18-25 years) to the Policy and Communication Evaluation (PACE) Vermont Study. Facebook and Instagram advertising produced the greatest number of survey starts (n=2013), followed by posts to a state-wide web-based neighborhood forum (n=822) and Google advertisements (n=749). Retention was 78.11% (1185/1517) at 3 months and 72.18% (1095/1517) at 6 months. Retention was equivalent across all incentive study conditions at both waves, despite a strong stated preference among study participants for the guaranteed payment at baseline. Youth had greater retention than young adults at both waves (wave 2: 395/480, 82.3% vs 790/1037, 76.18%; wave 3: 366/480, 76.3% vs 729/1037, 70.30%). Substance use prevalence in this cohort was similar to national and state-level surveillance estimates for young adults, but was lower than state-level surveillance estimates for youth. Most participants retained at wave 3 provided positive qualitative feedback on their experience.

**Conclusions:**

Our study supports the feasibility of recruiting a web-based cohort of youth and young adults with representation across an entire state to evaluate substance use prevention efforts. Findings suggest that a guaranteed payment immediately upon survey completion coupled with a bonus for completing all survey waves and weekly survey reminders may facilitate retention in a cohort of youth and young adults.

## Introduction

Adolescence and young adulthood are defined by developmental processes that mark increased susceptibility to risk-taking behaviors, including substance use [1-4]. In tobacco control, prevention efforts have shifted from individual and group-level interventions to population-based approaches, including policy and mass media efforts to reduce the appeal and accessibility of tobacco products to young people [5]. Concurrently, state-level cannabis policies in the United States have aimed to liberalize the accessibility of cannabis products, though there have been few state-level prevention campaigns. Using national surveillance data across states has been the standard approach to evaluate the effects of these policies on youth and young adult perceptions and behaviors [6,7]. These evaluations, which use cross-sectional data over time, may mask more nuanced trends in individual-level changes in harm perceptions and behavior and have largely failed to address spillover effects on other substance use. Novel surveillance methods that follow individuals over time and capture awareness of substance use prevention policy and communication efforts may provide a stronger basis for their evaluation.

Vermont represents a unique test case for the evaluation of population-level interventions for substance use for three reasons. First, the prevalence of substance use in young people from Vermont is higher than national estimates [8-10]. Second, Vermont has implemented a number of new policies related to substance use in the past 5 years, including a state-wide opioid drug disposal program, stronger prescribing guidelines, and requirements in the Vermont Prescription Monitoring System (2016); legalized possession of cannabis for adults aged 21 years or above [11] (2018); a ban on the web-based sale of e-cigarettes [12] and a 92% tax on e-cigarettes [13] (2019); and an increase in the legal age of tobacco sale to 21 years [14] (2019). In addition to policy efforts, the Governor’s 2018 Opioid Coordination Council Report recommended development and implementation of school-based primary prevention programs for opioid use and a comprehensive drug prevention messaging campaign [15]. Third, while Vermont has a relatively homogeneous population in terms of race/ethnicity, it is the second most rural state in the country [16], with between 60% and 100% of the area classified as rural depending on the definition [17]. Thus, policy and communication interventions may be implemented or experienced differently than in settings with greater population density.

In 2018, researchers and program staff at the University of Vermont and the Vermont Department of Health began discussing the development of a longitudinal cohort study of youth and young adults to evaluate responses to changes in tobacco, alcohol, and other substance use policies, communication, and interventions at the state level. The Policy and Communication Evaluation (PACE) Vermont Study was designed to complement existing evaluation efforts that rely on a combination of state-level surveillance and smaller convenience samples [18] The PACE Vermont Study uses web-based data collection in a large sample of youth and young adults, with surveys at shorter intervals to capture changes over time. The survey instrument was also designed to be flexible, allowing for assessment of emerging issues and communication outcomes (eg, knowledge, attitudes, and beliefs) not typically captured in state surveillance systems.

Web-based data collection was proposed to reduce barriers to participation in research among rural people from Vermont, given that as of 2018, 98% of young adults aged 18 to 29 years use the internet, as do 78% of adults who live in rural communities [19]. Similarly, 94% of young adults aged 18 to 29 years and 65% of adults in rural communities own a smartphone [20]. Adoption of web-based surveys for data collection is likely to appeal to young people, who grew up with computers and use them in virtually all aspects of their lives, while reducing transportation and other costs that serve as barriers to engaging in traditional clinical trials [21-24]. With respect to retaining young people in longitudinal studies, there was no clear recommendation on incentives to maximize retention, as previous studies had identified multiple means, including increasing participant payments, conducting sweepstakes, providing bonuses, and sending reminder postcards [25-28].

This study had the following two primary goals: (1) to identify the combination of recruitment strategies that would provide a sample of youth and young adults sufficiently representative of the Vermont population and (2) to determine how best to retain a web-based panel of youth and young adults to be able to attribute changes in knowledge, attitudes, beliefs, and behaviors to specific interventions. Specifically, this study experimentally compared the effects of a lottery payment, a guaranteed payment, and participant preference for a particular completion incentive on retention at 3-month and 6-month follow-ups. Our a priori hypothesis was that retention would be higher in the participant preference incentive condition than in the lottery or guaranteed payment incentive condition, as providing choice for some study-related decisions has been described as a means to improve retention in studies involving young adults [29].

## Methods

### Study Overview

The study consisted of three web-based surveys conducted from March 2019 through October 2019 and was approved by the Institutional Review Boards of the University of Vermont and Vermont Department of Health. This research also received a Certificate of Confidentiality from the National Institutes of Health.

### Recruitment and Enrollment

Eligible participants were Vermont residents aged 12 to 25 years who were willing to complete three 10- to 15-minute web-based surveys over a 6-month period. Youth participants aged 12 to 17 years also had to report being a US citizen or permanent resident. Recruitment was conducted by Hark, a Vermont-based digital design and marketing firm [30], over a 10-week period (March 26-June 4, 2019). Participants were recruited via the following three main mechanisms: (1) web-based recruitment including both paid and unpaid advertising, (2) community recruitment through partner organizations, and (3) participant referrals via a personalized link. Each recruitment type contained a unique link to the study website to be automatically tracked via Google Analytics. Web-based recruitment occurred through paid Facebook, Instagram, and Google display and Gmail advertisements; free Craigslist Vermont advertisements; posts on PACE Vermont’s social media accounts (Facebook, Instagram, and Twitter); paid posts on a state-wide online neighborhood forum (Front Porch Forum) [31]; and paid placement of advertisements in local web-based and print newspapers with relevant news stories. Community recruitment occurred through outreach from engaged partners to their constituents and via news media on the PACE Vermont Study. Partner organizations received a recruitment toolkit, with a tailored newsletter blurb, flyers, sample email language, and sample social media posts for print and digital promotion of the study, as well as a timeline for distribution of recruitment material to their networks. Participant referrals were requested at the end of baseline survey completion via a thank you email with a personalized referral link, a request to share the survey link with friends on social networks, and a direct link to the PACE Vermont Facebook page. Parents of youth participants were able to opt in to be considered for a small study incentive (US $5) for referring another eligible family to the study using their unique link. The PACE research team met weekly to monitor representation by age, race, ethnicity, and county, and promotional strategies during this period were adjusted to focus on underrepresented areas of the state or sociodemographic groups.

Digital advertising channels, including Google display advertisements, Google Gmail advertisements, and Facebook display advertisements, delivered content to the following three Vermont-based segments: youth (aged 12-17 years), young adults (aged 18-25 years), and parents (aged ≥18 years). In addition to age and geography, Google and Facebook’s interest and lifestyle-based targeting enabled specific targeting of parents. Prior to recruitment, the PACE team integrated Google and Facebook advertisements with the study landing page and its web analytics tool. Google and Facebook campaigns for the PACE Vermont Study were set to optimize their targeting algorithms and advertisement variations to achieve a maximum number of survey starts rather than clicks or page views. The initial structure of both the Facebook and Google advertisement campaigns mirrored the audience segments (youth, young adults, and parents). Advertisements for each segment featured visual assets (ie, photographs, illustrations, and graphics) and text in the form of headlines, posts, email body, and call-to-action buttons. Advertisements directed at youth and young adults used the following message themes: (1) earn cash rewards and buy the things you really want and (2) be a leader in your community and share your opinion on important topics. Advertisement visuals took the following two approaches: (1) photographs of youth and young adults smiling and holding a mobile phone or gift card and (2) eye-catching illustrations and graphics with action-oriented phrases like “We want to hear from you.” Advertisements directed at parents were designed to motivate parents to encourage their children to join the study. Parent-focused messages used the following themes: (1) your family can help improve the health of Vermont’s youth for years to come and (2) your teen’s participation will help Vermont create substance-abuse resources for other families. Advertisement visuals took the following two approaches: (1) humorous photography of toddlers and young children making messes and (2) sentimental photography of parents embracing their babies and young children. A separate wave of Facebook and Google advertisements ran close to the end of the recruitment window and highlighted the urgency of participation with messages like “Don’t miss out on PACE VT” and “Time is running out.” Other promotional channels like Front Porch Forum and Craigslist primarily targeted parents and older young adults. Because these channels do not allow for advanced targeting beyond geography, promotional messages mirrored those of parents in our paid campaigns.

All study advertisements and links directed participants to the PACE Vermont website, where there was a brief study description and link to an open web-based screener. In addition to direct advertising to youth, parents of eligible youth were also targeted via promotional efforts. They were asked to review an information sheet and provide informed consent prior to youth providing assent to complete the screener. Youth who initiated the screening survey without parental consent were asked to provide parent contact information, which triggered an email to the parents. Upon parental consent, youth received a unique link to the screener to provide assent. Youth and young adults underwent an electronic informed consent process and received an email link to contact study personnel to ask questions about the study. Eligible consenting youth and young adults were automatically forwarded to the baseline survey, where they completed demographic information and questions on substance use knowledge, attitudes, beliefs, and behaviors. These included measures of ever and past 30-day use of cigarettes, alcohol, and marijuana, which served as benchmarks for comparison to prevalence estimates from state and national surveillance on substance use data. Participants were then automatically forwarded to a web-based version of the University of Vermont participant payment form required for internal tracking of study payments. All surveys were voluntary, deployed via Qualtrics [31], and optimized for completion via a computer or mobile phone.

During baseline data collection, we revised our survey delivery to ensure the eligibility of participants and the validity of study responses in several ways as follows: (1) adding automatic screening within our survey platform, Qualtrics [32], to exclude participants with an IP address outside Vermont; (2) conducting consistency checks between age and date of birth, as well as state of residence and location of IP address; (3) adding a CAPTCHA item in the screener to ensure that respondents were human and not bots; (4) conducting additional screening of respondents with suspicious email addresses (eg, common e-mail format across surveys completed within minutes of each other and email addresses including names that did not correspond to contact information) and out-of-state phone numbers; and (5) using information from the screening and payment forms (eg, consistency of name across forms and location of participant address) to verify eligibility. Potentially fraudulent participants were flagged and received an email from the study team offering an opportunity to confirm their contact information and remain in the study. Respondents who did not confirm that they were valid participants were removed from the study.

### Intervention and Retention

At the end of the baseline survey, participants were asked “Which of the following would you like to receive for completing other web-based surveys like this?” with the following two response choices: “Receive a $10 online gift card” or “Be entered into a lottery to receive $50.” After responding to this question, participants were randomly assigned within the Qualtrics survey system to one of the following three study conditions: (1) guaranteed incentive (US $10), (2) lottery incentive (US $50 weekly lottery drawing), and (3) preferred method (guaranteed or lottery, based on the response to the question). Participants were informed of their study condition, and those in the guaranteed condition were automatically directed to a web interface [33] to confirm their email address and receive their US $10 electronic gift card (wave 1 study payment). Gift cards were emailed to wave 1 participants once they were confirmed as participants with valid completion. Lottery drawings were conducted among those who had completed the survey in a given week (n=3 youth and n=3 young adult winners selected each week during each survey wave), and winners were notified by email. Participants were told that they would receive a bonus payment for completing all three surveys (lottery payment group: US $50 bonus; guaranteed payment group: US $20 bonus in addition to US $10 per completed survey). Thus, all participants received US $50 upon completing all three surveys and lottery participant winners could receive more.

Follow-up surveys were launched approximately 3 months (June 27-July 31, 2019) and 6 months (September 17-October 15, 2019) after the baseline survey. Each survey was distributed initially via email or text message, based on participant preference, with a message that notified participants about the dates of data collection, including the 1-month window during which the follow-up survey would be open for completion. Weekly reminder messages were sent to youth and young adults who had not completed the survey via both email and text message throughout the 1-month window, with additional reminders in the last 2 days of each window via email, text message, and social media. Incentive payments at each follow-up were dictated by the study condition, with those in the guaranteed condition automatically linked to Rybbon to confirm their email address and receive their US $10 gift card immediately. As in the baseline survey, lottery drawings (US $50) were conducted each week during data collection for the two follow-up surveys and winners were notified by email. At the completion of the final follow-up survey, participants who had completed all three waves received their bonus payment immediately via Rybbon.

### Participant Feedback

At the end of the final survey, participants were asked the following two questions about their experience in the PACE Vermont Study: “What was your favorite part of participating in the PACE Vermont Research Study?” and “What could we improve in the PACE Vermont Research Study to make it easier for you to participate?” Responses were open-ended. Two coders (SEL and CM) reviewed the responses and created in vivo inductive categories from themes that arose during the course of data analysis, as described by Miles and Huberman [34]. Responses for each question were then coded in NVivo software (QSR International) independently by each coder, and responses were allowed to fall into more than one category. Reliability of the coders for each category ranged from a kappa value of 0.47 to 0.97, representing moderate to almost perfect agreement. For the question “What was your favorite part of participating in the PACE Vermont Research Study?” the coding category with the lowest level of agreement was “other” (κ=0.47) and the category with the highest level of agreement was “compensation” (κ=0.97). For the question “What could we improve in the PACE Vermont Research Study to make it easier for you to participate?” the coding category with the lowest level of agreement was “learning” (κ=0.54) and the category with the highest level of agreement was “compensation” (κ=0.88).

### Statistical Analysis

This study used the following two sources of data: advertising metrics, and enrollment and follow-up data from our surveys. First, we estimated the cost per survey start according to the recruitment source by dividing the total amount spent on each source by the number of survey starts. Second, we developed a CONSORT diagram to track participants from enrollment through the three survey waves and estimated differences in retention by the intervention condition at each stage using chi-square tests. We conducted additional analyses to test whether retention differed by concordance between preference and the intervention condition (ie, lottery vs guaranteed compensation). We also examined the distribution of sociodemographic characteristics (ie, county, sex, race/ethnicity, and employment status) and substance use (ever and past 30-day cigarette, alcohol, and marijuana use) by age and the intervention condition using chi-square tests and *t* tests and the differences in these characteristics among those retained at all three waves versus those lost to follow-up. Survey weights were developed post-hoc from population estimates of females and males between the ages of 12 and 25 years (year by year) residing in each of Vermont’s 14 counties in 2017 [35]. The goal of survey weighting was to determine how closely the convenience sample matched other state surveillance, as well as correct for the higher response by females and those residing in Chittenden County. Each cell of the table was divided by the total number of individuals between the ages of 12 and 25 years residing in the 14 counties (n=116,407) to generate population-based proportions. A comparable table of survey respondents was created, totaling the number of individuals who completed the baseline survey by sex, age, and county of residence. Each subtotal was divided by the total number of respondents (n=1517) to generate sample-based proportions. Survey weights were then calculated by dividing population proportions by sample proportions, again by sex, age, and county. We compared the weighted prevalence of ever and past 30-day use of cigarettes, alcohol, and marijuana in our sample to national and state-level surveillance estimates from the National Survey on Drug Use and Health (NSDUH) [10,36]. Finally, we assessed the major categories of responses to two participant feedback items from qualitative coding.

## Results

### Recruitment and Enrollment

Table 1 presents information on new visitors to the study website; survey starts for parents, youth, and young adults; and cost per survey start by recruitment source. Survey starts by recruitment source were tracked using Google Analytics on the PACE Vermont study website. Survey completions by recruitment source could not be captured as surveys were conducted within Qualtrics. Overall, there were 9975 new visitors to the study website, with Facebook and Instagram advertisements accounting for 54.55% (5441/9975) of web traffic, with 2013 survey starts. Google display and Gmail advertisements accounted for 35.10% (3501/9975) of web traffic and 749 survey starts. Three postings in an email digest with state-wide coverage (Front Porch Forum) generated 9.75% (973/9975) of web traffic and 939 survey starts. Partner referrals, newspaper print advertisements, and Craigslist advertisements produced smaller numbers of new users and survey starts. The cost per conversion to a survey start was US $382 considering all recruitment sources; when newspaper print advertisements were excluded, the cost per conversion to a survey start was US $11. Only four parents received the US $5 referral incentive.

The best performing Google advertisements were Gmail advertisements run close to the end of the recruitment window highlighting the urgency of participation for parents, youth, and young adults; the top Facebook posts for youth and young adults highlighted urgency and the importance of youth and young adult feedback (“We want to hear from you.”) The top performing Facebook post for parents cited how findings from the PACE Vermont Study would be used to guide resources for substance use prevention in Vermont. While advertisements targeting youth ran for the duration of the recruitment period, advertisements targeting parents were more effective at recruiting youth than youth-specific advertisements.

**Figure 1:**
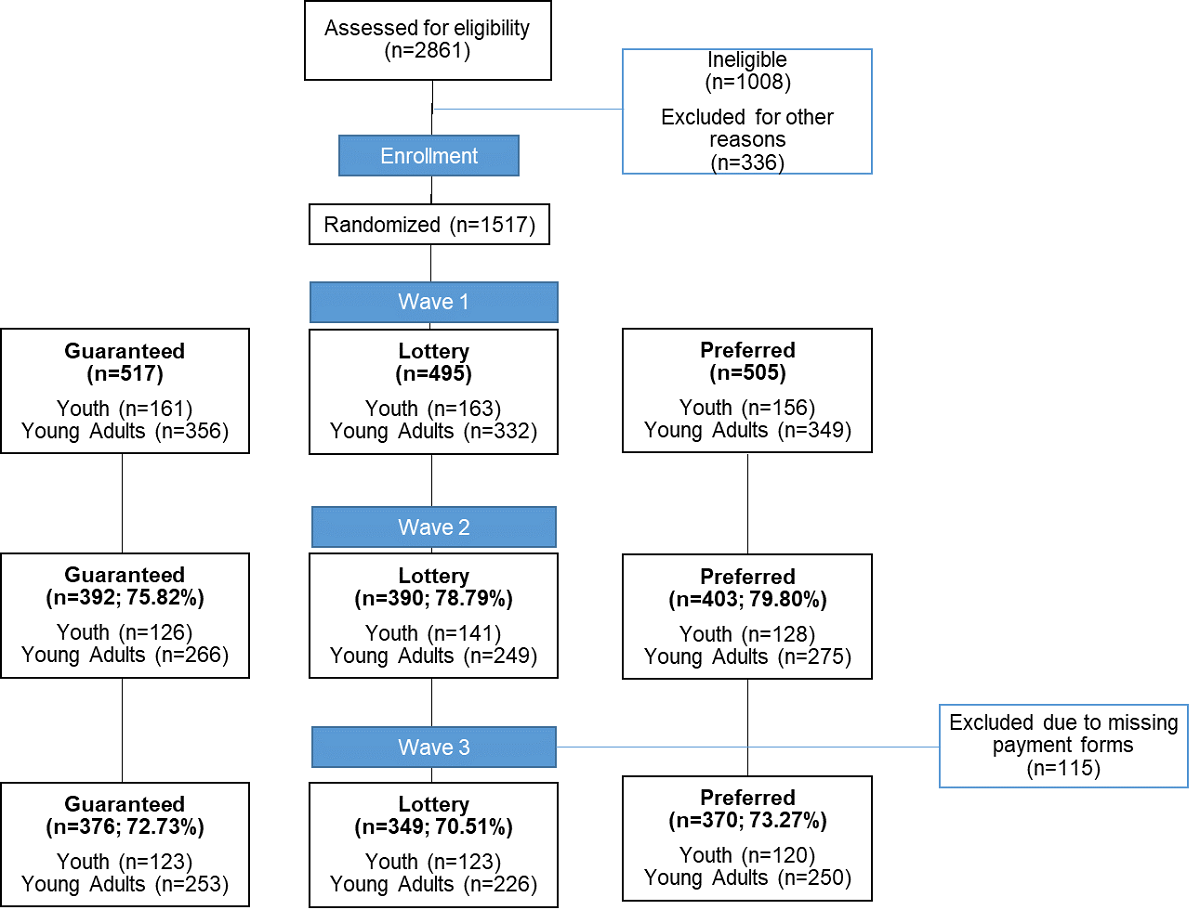
CONSORT flow diagram of participants in the Policy and Communication Evaluation (PACE) Vermont Study, 2019.

In addition to the 2723 youth and young adult survey starts from the study advertisements, participants referred others to the study, resulting in 2861 completed screenings (Figure 1). Of those assessed for eligibility, 1008 were deemed ineligible (eg, not aged 12-25 years and not Vermont residents) and 336 were excluded for other reasons, including being flagged as potentially fraudulent.

Overall, there were 480 youth respondents and 1037 young adult respondents included in the PACE Vermont Study sample (Table 2). The recruitment cost per eligible enrolled participant was US $29 (US $44,111/1517 participants). Participants represented each of the 14 counties in the state, with the distribution by county generally reflecting 2017 population estimates for Vermont youth and young adults [35]. The majority of the sample was female (1071/1517, 70.60%), was white (1318/1517, 86.88%), and reported working either part-time or full-time (898/1517, 59.20%).

Table 3 presents the number of responses and weighted prevalences of cigarette, alcohol, and marijuana use by age group in the PACE Vermont Study compared with estimates for the same measures in the NSDUH. Ever use estimates for each substance were provided in the 2018 NSDUH national report [36] and past 30-day measures were drawn from the 2016-2017 state-level report [10]. Prevalence of ever cigarette, alcohol, and marijuana use in PACE Vermont Study youth participants was generally similar to national estimates from the NSDUH, although past 30-day use estimates tended to be lower in PACE Vermont youth compared with state-level estimates from the NSDUH. Ever and past 30-day use in young adult PACE Vermont participants tracked closely with NSDUH estimates, with the following three exceptions: past 30-day cigarette use was lower in the PACE Vermont sample compared with the NSDUH estimate (18.78% vs 33.38%), and the PACE Vermont sample showed higher ever alcohol use (PACE vs NSDUH: 89.52% vs 79.70%) and ever marijuana use (70.57% vs 51.50%).

**Table 1:** Recruitment sources, costs, and conversions in the PACE Vermont Study 2019.

Source	Cost (total: US $44,110.71)	New users (N=10,250)	Conversions, n				Cost per conversion^a^		
			Parent survey starts (N=1026)	Young adult survey starts (N=1772)	Youth survey starts (N=951)	Total survey starts (N=3749)			
Facebook & Instagram advertising	US $23,676.42	5441	443	1247	323	2013		US $11.76	
Google display & Gmail advertising	US $9213.78	3501	331	233	185	749		US $12.30	
Front Porch Forum (paid post, partial state coverage)	US $4950.00	223	37	26	54	117		US $42.31	
Front Porch Forum (two sponsored posts, state wide^b^)	US $3666.00	1025	199	248	375	822		US $4.46	
Newspaper print advertisements	US $2604.51	1	1	0	0	1		US $2604.51	
Craigslist	US $0.00	8	1	4	0	5		US $0.00	
Partner sources	US $0.00	51	14	14	14	42		US $0.00	

^a^Average cost per conversion was US $381.55, and average cost per conversion, excluding print advertisements, was US $11.06.

^b^Sponsored posts available to the Vermont Department of Health for outreach activities.

**Table 2:** Participant characteristics by incentive condition in the PACE Vermont Study 2019.

Characteristic^a^		Incentive condition			Total (N=1517), n (%)	*P* value
		Guaranteed (N=517), n (%)	Lottery (N=495), n (%)	Preference (N=505), n (%)		
**Age group (years)**						.75
	12-17	161 (31.14)	163 (32.93)	156 (30.89)	480 (31.64)	
	18-25	356 (68.86)	332 (67.07)	349 (69.11)	1037 (68.36)	
**County of residence**						.42
	Addison	41 (7.93)	33 (6.67)	40 (7.92)	114 (7.51)	
	Bennington	20 (3.87)	18 (3.64)	16 (3.17)	54 (3.56)	
	Caledonia	16 (3.09)	23 (4.65)	21 (4.16)	60 (3.96)	
	Chittenden	228 (44.10)	225 (45.45)	203 (40.20)	656 (43.24)	
	Essex	—^b^	—	8 (1.58)	14 (0.92)	
	Franklin	33 (6.38)	25 (5.05)	25 (5.95)	83 (5.47)	
	Grand Isle	6 (1.16)	5 (1.01)	—	15 (0.99)	
	Lamoille	10 (1.93)	19 (3.84)	25 (4.95)	54 (3.56)	
	Not sure	9 (1.74)	10 (2.02)	12 (2.38)	31 (2.04)	
	Orange	14 (2.71)	20 (4.04)	12 (2.38)	46 (3.03)	
	Orleans	10 (1.93)	8 (1.62)	11 (2.18)	29 (1.91)	
	Rutland	22 (4.25)	24 (4.85)	37 (7.33)	83 (5.47)	
	Washington	68 (13.15)	51 (10.30)	62 (12.28)	181 (11.93)	
	Windham	18 (3.48)	18 (3.64)	18 (3.56)	54 (3.56)	
	Windsor	19 (3.68)	12 (2.42)	11 (2.18)	42 (2.77)	
**Sex**						.16
	Female	356 (68.86)	361 (72.93)	354 (70.10)	1071 (70.60)	
	Male	161 (31.14)	132 (26.67)	151 (29.90)	444 (29.27)	
**Race/ethnicity**						.21
	White	442 (85.49)	444 (89.70)	432 (85.54)	1318 (86.88)	
	Nonwhite/other/multiple race	45 (8.70)	34 (6.87)	45 (8.91)	124 (8.17)	
	Hispanic	30 (5.80)	16 (3.23)	28 (5.54)	74 (4.88)	
**Employment status**						.33
	Do not currently work for pay	222 (42.94)	207 (41.82)	189 (37.43)	618 (40.74)	
	Work part-time (<15 hours/week)	101 (19.54)	80 (16.16)	106 (20.99)	287 (18.92)	
	Work part-time (15-34 hours/week)	74 (14.31)	83 (16.77)	85 (16.83)	242 (15.95)	
	Work full-time (35 hours/week or more)	120 (23.21)	124 (25.05)	125 (24.75)	369 (24.32)	

^a^There were missing data for county (n=1), sex (n=2), race/ethnicity (n=1), and employment status (n=1).

^b^Suppressed due to unweighted numerator <5 or unweighted denominator <50.

**Table 3:** Comparison of substance use prevalence by age group in the PACE Vermont sample and the National Survey on Drug Use and Health.

Characteristic		Youth (aged 12-17 years)						Young adults (aged 18-25 years)					
		Value, n		Weighted % (95% CI)		NSDUH^a^ estimate		Value, n		Weighted % (95% CI)		NSDUH estimate	
**Cigarette use**													
	Ever		42		9.1% (6.3-12.9)		9.6% [36]^b^		487		47.4% (43.4-51.4)		45.9% [36]^b^
	Past 30 days		11		2.2% (1.1-4.4)		5.8% [10] (4.5-7.3)		178		18.8% (15.9-22.1)		33.4% [10] (28.9-38.2)
**Alcohol use**													
	Ever		141		29.4% (24.5-34.7)		26.3% [36]^b^		935		89.5% (86.9-91.7)		79.7% [36]^b^
	Past 30 days		43		9.3% (6.6-12.9)		13.6% [10] (11.3-16.3)		743		70.8% (66.9-74.3)		70.9% [10] (66.7-74.8)
	Binge alcohol use, past 30 days		13		3.1% (1.6-5.8)		7.2% [10] (5.7-9.0)		484		48.3% (44.3-52.3)		49.3% [10] (44.8-53.7)
**Marijuana use**													
	Ever		80		16.3% (12.8-20.7)		15.4% [36]^b^		742		70.6% (66.7-74.1)		51.3% [36]^b^
	Past 30 days		47		8.7% (6.3-12.1)		10.8% [10] (8.7-13.2)		412		41.3% (37.4-45.3)		38.8% [10] (34.2-43.7)

^a^NSDUH: National Survey on Drug Use and Health.

^b^95% CI not provided for NSDUH national estimates of ever use.

### Intervention and Retention

Randomization of the 1517 eligible participants was generally equal across study conditions, with 34.08% (517/1517) allocated to the “guaranteed” incentive, 32.63% (495/1517) to the “lottery” incentive, and 33.29% (505/1517) to the “preferred” incentive. Proportions were similar when looking at youth and young adult subgroups separately. There were no differences in the distribution of participants to the study condition by age, county of residence, sex, race, ethnicity, or employment status (Table 2). Response to the question about incentives for completing similar web-based surveys indicated a strong preference for the guaranteed incentive (1304/1517, 85.96%), and 67.96% (1031/1517) of participants were assigned to an incentive condition concordant with their preference.

At wave 2, 78.11% (1185/1517) of the full sample completed the survey. Youth retention at wave 2 was 82.3% (395/480), while young adult retention was 76.18% (790/1037; *P*=.007). At wave 3, 72.18% (1095/1517) of the full sample completed the survey; again, youth had higher retention (366/480, 76.3%) than young adults (729/1037, 70.30%; *P*=.02). Overall, 70.20% (1,065/1517) completed all three waves of the study (74.0% [355/480] of youth and 68.47% [710/1037] of young adults; *P*=.03).

Retention did not differ by incentive condition at wave 2 (*P*=.28) or wave 3 (*P*=.59). Retention was also similar when examining concordance between preference and the intervention condition, with 77.98% (804/1031) retention at wave 2 among those with concordant preference and an incentive condition compared with 78.4% (380/485) in the nonconcordant group (*P*=.87). Proportions were also similar at wave 3 (concordant: 751/1031, 72.84%; nonconcordant: 343/485, 70.7%; *P*=.39). Retention across all three waves did not differ by county, sex, race/ethnicity, or employment status. Retention was lower, however, among participants who had ever used a cigarette (*P*=.005) and those who reported past 30-day use of marijuana (*P*=.001; data available upon request).

### Participant Feedback

Of the 1095 respondents in the wave 3 survey, 86.58% (948/1095) provided a response to the question regarding their favorite part of the survey and 81.55% (893/1095) provided a response to the question regarding potential improvements to the survey. Table 4 presents coded responses to each item. With some responses categorized into more than one code, there were 1049 responses to the item on the favorite part of participating in the study, with 28.60% (300/1049) noting financial compensation as their favorite part, followed by learning something new (192/1049, 18.30%) and making a meaningful contribution to science or to the community (184/1049, 17.54%). There were 910 responses to the item on potential improvements to the study, with the majority noting no changes needed (496/910, 54.5%), followed by specific recommendations related to ease of use (302/910, 33.2%). Recommendations included suggestions for improvements to survey structure, survey timing, and survey wording.

**Table 4:** Participant feedback at the end of the PACE Vermont Study.

Question, code, and subcode			Value, n (%)
**What was your favorite part of participating in the PACE^a^ Vermont Research Study? (1049 responses)**			
	Survey task (κ=0.50) *Favorite part was the survey task itself.*		162 (15.4)
	**Learning (κ=0.57)** ***Favorite part was learning something new.***		192 (18.3)
		Learning: self (κ=0.91) *Enjoyed learning something about themselves or having an opportunity to self-reflect.*	85 (8.1)
		Learning: other (κ=0.72) *Enjoyed learning new information about policies, organizations, or substances.*	89 (8.5)
	Ease of use (κ=0.80) *Favorite part was the ease of use and accessibility of the surveys.*		110 (10.5)
	Contribution (κ=0.83)** * Favorite part was making a meaningful contribution to something (eg, science and the community).*		184 (17.5)
	Compensation (κ=0.97) *Favorite part was the financial compensation.*		300 (28.6)
	Other (κ=0.47) *Response not otherwise categorized.*		80 (7.6)
	None (κ=0.71) *Did not generate a response (eg, “I did not have a favorite part”).*		21 (2.0)
**What could we improve in the PACE Vermont Research Study to make it easier for you to participate? (910 responses)**			
	Learning (κ=0.54) *Requested more resources or opportunities to learn.*		16 (1.8)
	**Ease of use (κ=0.88)** ***Suggested improving survey’s ease of use.***		302 (33.2)
		Survey design (κ=0.81)** * Suggested improvement to survey structure or design.*	119 (13.1)
		Reminders & timing (κ=0.87)** * Suggested improvement to timing of surveys or the reminder system.*	43 (4.7)
		Question improvement (κ=0.84) *Suggested improvement to wording or content of survey questions.*	140 (15.4)
	Compensation (κ=0.88) *Suggested improvement to the compensation system.*		53 (5.8)
	Other (κ=0.58)** * Not otherwise categorized.*		43 (4.7)
	**None (κ=0.78)** *** No response generated (eg, “nothing”).***		496 (54.5)
		None: positive (κ=0.83) *Response generated was wholly positive.*	244 (26.8)

^a^PACE: Policy and Communication Evaluation.

## Discussion

### Principal Findings

This study identified successful recruitment strategies for a web-based cohort study of youth and young adults to inform and evaluate state-level substance use prevention efforts. It also tested the effect of three incentive conditions (guaranteed, lottery, and preferred) on retention over a 6-month period. Over a 10-week period in 2019, we were able to recruit 480 eligible youth and 1037 eligible young adults to the PACE Vermont Study. Findings from this study indicated that Facebook and Instagram advertising produced the greatest number of survey starts, followed by posts to a state-wide online neighborhood forum in Vermont (Front Porch Forum) and Google advertisements (display and Gmail). The integration of Google and Facebook advertisements with the study landing page and its web analytics tool was critical to evaluating survey starts as the advertising metric of interest in support of PACE’s recruitment efforts. Validation of study responses was achieved through multiple methods, in line with other web-based studies [37]. Data collected on county supported the distribution of responses across the state, in line with the distribution of youth and young adults in the population. The success of the local online neighborhood forum in driving traffic to the study website highlights the potential importance of these venues in recruitment. While community partner sources did not drive the same level of traffic to the site, advertisement of the study via these community organizations and through the local web-based digest may have lent credibility to the study and increased awareness that improved recruitment. Substance use prevalence estimates in PACE Vermont youth and young adults generally tracked national estimates from the NSDUH, although youth reported lower prevalence of current cigarette, alcohol, and marijuana use than estimates from NSDUH’s state-level surveillance.

Retention was 78.11% (1185/1517) at 3 months and 72.18% (1095/1517) at 6 months. Contrary to our hypothesis, retention was equivalent across all incentive study conditions. This may have been due to a strong stated preference among study participants for the guaranteed payment and assignment of approximately two-thirds of participants to an incentive condition that was concordant with their preference. Youth participants at both waves had greater retention than young adult participants. Participants retained at all three waves were less likely to be ever cigarette users or past 30-day marijuana users compared with those lost to follow-up. The majority of participants retained at wave 3 provided feedback on their experience of the study, with largely positive comments about compensation, learning something new, and making a difference. Participants also provided specific feedback to improve future surveys, such as requests for more resources or opportunities to learn and suggestions to improve timing and reminders for surveys.

### Limitations

The PACE Vermont Study was limited to a small largely rural state, and thus, successful recruitment strategies may not be generalizable to other study contexts. Additionally, while the sample was generally aligned with population distribution by county, there were imbalances by sex in the study sample, with the majority of the sample being female (1071/1517, 70.60%) in contrast to 48% of the state population of youth and young adults. There was also one county (Chittenden) with a higher response than expected according to population distribution, but this county is both the most populous and home to the University of Vermont with the largest population of undergraduates in the state who would have been eligible for the study. Lower prevalence of past 30-day cigarette, alcohol, and marijuana use in the PACE Vermont youth compared with state estimates suggests that youth enrolled in this study may represent a lower risk sample. This may be due to the recruitment process for youth that required parental consent to participate, thus attracting a lower risk pool of youth, or the smaller sample size of youth than young adults. Additional attrition by ever cigarette users and past 30-day marijuana users may have produced a sample with fewer risk behaviors at follow-up. Given the randomized nature of the incentive condition and balance in demographic characteristics across conditions, findings related to retention are likely to be generalizable to other web-based survey studies of youth and young adults.

### Comparison With Prior Work

Similar to other web-based studies [28,38], Facebook and Instagram advertisements provided the greatest reach and lowest cost per survey start when considering parent, youth, and young adult screener surveys; these were followed by Google advertisements via display and Gmail. The success of these strategies may have been related to consistency with recommendations for Facebook recruitment, including having an attractive website [18] and existing social media accounts (@pace_vt) that identified the partner organizations involved in the study (ie, University of Vermont and Vermont Department of Health) and supported the credibility of the study [34].

Retention in our cohort of youth and young adults was higher at 6 months (72.18%) compared with a national cohort of young adults aged 15 to 21 years who also completed web-based surveys (63%) [27]. Our randomized experiment regarding incentive conditions showed, similar to previous studies [37-40], that multiple means of compensation produce equal retention when combined with providing completion bonuses and sending multiple reminders. However, our baseline item regarding incentive preference suggests that the majority of youth and young adults prefer a small guaranteed payment for responding to a web-based survey. Thus, providing guaranteed compensation could help to improve recruitment of youth and young adults in future studies. Our ongoing retention efforts draw on expertise gathered from in-person cohort studies of youth and young adults [25,29]; we send birthday postcards to participants at the start of each month and continue to post on our social media accounts to retain awareness of and engagement in the study.

### Conclusions

Findings from the PACE Vermont Study demonstrated the feasibility of using traditional web-based advertising strategies (eg, Facebook and Google), in addition to web-based outreach through local community forums and organizations, to recruit a cohort of 1517 Vermont youth and young adults for a web-based study to evaluate state-level substance use prevention efforts. Participants were well distributed by age and county according to state population estimates and reported substance use prevalence comparable to national estimates in these age groups. Youth participants generally reported lower prevalence of risk behaviors compared with state-level estimates [10]. The higher proportion of female participants enrolled in the PACE Vermont Study is consistent with other studies, documenting higher recruitment of women to health studies via web-based advertising [38,40,41]. Retention in this web-based cohort study was over 70% at a 6-month follow-up and did not differ by the incentive condition, as seen in other studies of young adults [27]. Participant feedback on the study experience was positive. Results from our study suggest that providing a guaranteed payment immediately upon survey completion coupled with a bonus for completing all survey waves and weekly survey reminders may facilitate retention in a cohort of youth and young adults. Future work in our cohort will assess the impact of other means of retaining participants, including ongoing cohort engagement via regular contact (eg, birthday postcards and social media posts), ongoing community engagement (eg, reporting to community partners on PACE Vermont Study outcomes), and nonfinancial incentives (eg, lottery for PACE-branded items).

## Abbreviations

NSDUH: National Survey on Drug Use and Health
PACE: Policy and Communication Evaluation
